# Usual Presentation Has Odds: Unilateral Tibial Hemimelia in One of Dizygotic Twins

**DOI:** 10.7759/cureus.12834

**Published:** 2021-01-21

**Authors:** Muath Mamdouh Mahmod Al-Chalabi, Wan Azman Wan Sulaiman

**Affiliations:** 1 Reconstructive Sciences Unit, Universiti Sains Malaysia School of Medical Sciences, Kubang Kerian, MYS; 2 Plastic and Reconstructive Surgery, Universiti Sains Malaysia School of Medical Sciences, Kubang Kerian, MYS

**Keywords:** hemimelia, tibial hemimelia, congenital tibial deficiency

## Abstract

Tibial hemimelia is a relatively rare congenital tibial longitudinal deficiency (approximately 1 per 1 million live births), unilateral or bilateral, with a relatively intact fibula. Hemimelia results from a disruption of the lower limb developmental field during embryogenesis due to slow or even abort of chondrification process, which results in leg length discrepancy. Affected leg commonly appears short and deformed with knee, ankle, and foot involvement. It may present with a variety of associated anomalies. Surgical treatment varies according to the type and degree of deformity, and reconstructive interventions are still limited. Reported cases of tibial hemimelia are very infrequent, especially tibial hemimelia in twins. Usually, the cases were in single embryo or less frequently in one of the monozygotic twins, but no reported cases regarding tibial hemimelia in one of the dizygotic twins as this article reports.

## Introduction

There is a wide range of congenital long-bone anomalies, including Amelia and Hemimelia. Amelia is defined as an absence of the entire extremity, while Hemimelia is the term used to describe a partial or complete congenital absence of a limb's distal half, as in radial, ulnar, tibial, and fibular hemimelia. Complete hemimelia is an extremity deficiency from the elbow or knee level with an absence of the distal elements. Paraxial (longitudinal) hemimelia is a deficiency of one of the forearm or leg bones extending into the extremity's most distal parts [1]. Tibial hemimelia is a rare congenital anomaly characterized by insufficiency of the tibia longitudinally with a comparatively intact fibula. The occurrence of congenital deficiency of the tibia is approximately 1 per 1 million live births [2-7]. The percentage of monozygotic twins is about 0.8%; therefore, the occurrence of tibial hemimelia in monozygotic twins is 1 case per 125 million [4]. The first reported case of tibia hemimelia was in 1861 [3]. Tibia hemimelia commonly appears as a short and deformed leg with knee and ankle involvement, and the foot will be in an abnormal position. The precise etiology of tibial hemimelia is still unclear. However, the families with possible autosomal dominant or autosomal recessive inheritance are reported in the literature [8]. But, still no clear evidence of X-linked inheritance [9]. In Hospital Universiti Sains Malaysia, we reported a rare case of 16-month-old baby presented with a unilateral left distal tibial hemimelia with equinovarus deformity in one of the dizygotic twins without a family history of this deformity. Moreover, in this case, the condition is associated with hypospadias, and one dominant artery in the distal left lower limb. As the reconstructive options are limited, tighten tendon released, and Z-Plasty performed to increase the foot angle.

## Case presentation

**Figure 1:**
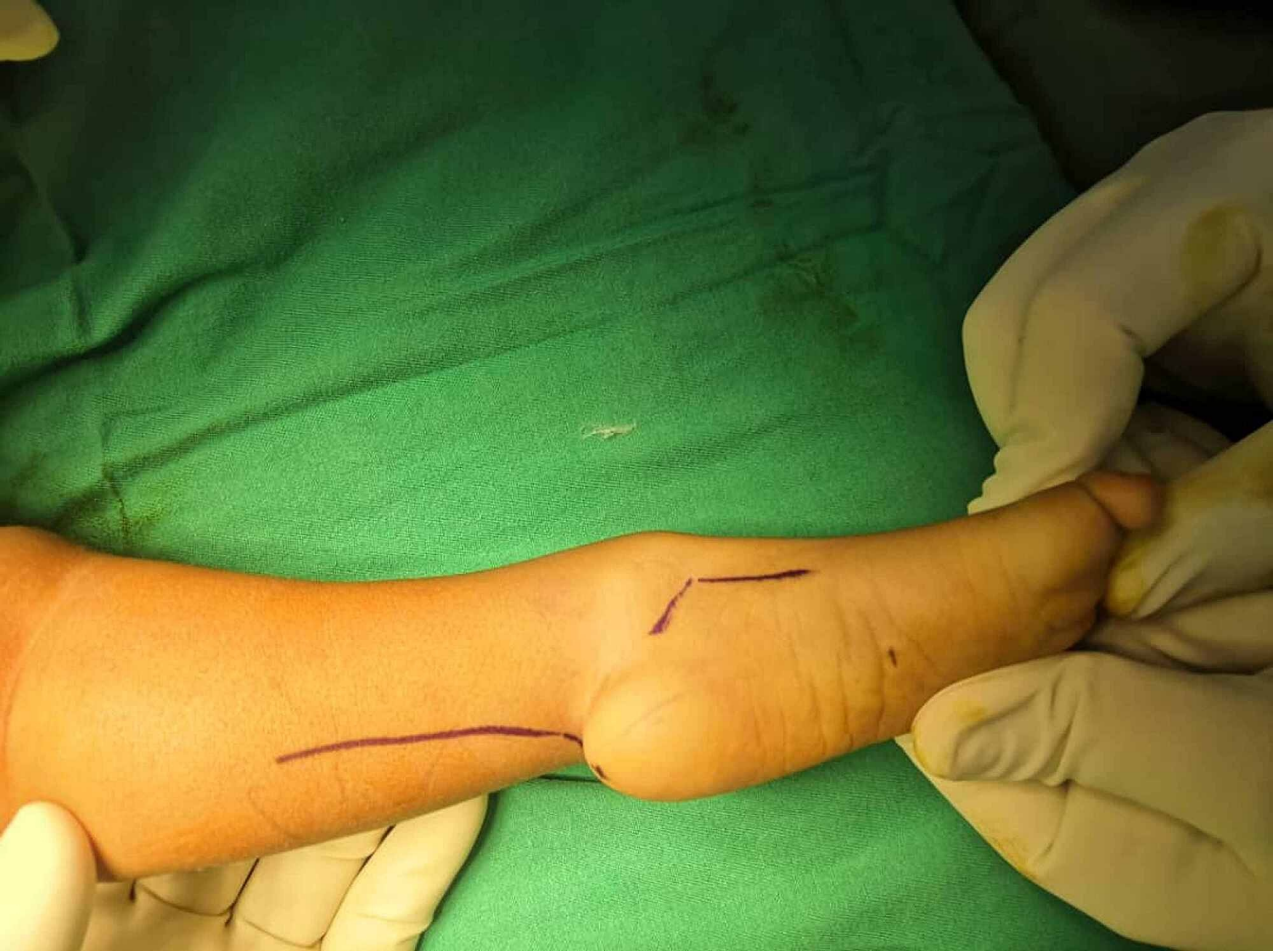
Left lower limb severe equinovarus deformity

We reported a 16-month-old pre-term baby boy who is a second of the dizygotic twins, born via emergency lower segment cesarean section due to breech presentation of presenting twin. A baby was diagnosed with unilateral left distal tibial hemimelia with a severe equinovarus deformity and one dominant artery in the distal left lower limb. The mother passed through uneventful pregnancy without any comorbidity, and there is no consanguinity with her husband. A family history of a similar congenital anomaly was negative. On examination, the baby was not dysmorphic with intact lip and palate; glans hypospadias with minimal chordae is associated comorbidity. The hip is stable, and the right lower limb completely normal with intact movement. Left limb anterolaterally bowed with shortening of the left tibia. Left fibula head and proximal tibia are palpable. The patient can extend a left knee actively but knee flexion up to 100 degrees. The left foot is small, supinated, and medially rotated with severe equinovarus deformity, as shown in Figure 1. The patient cannot dorsiflex the left foot but can extend and flex the left toes, although a big toe is underdeveloped.

Investigations

**Figure 2:**
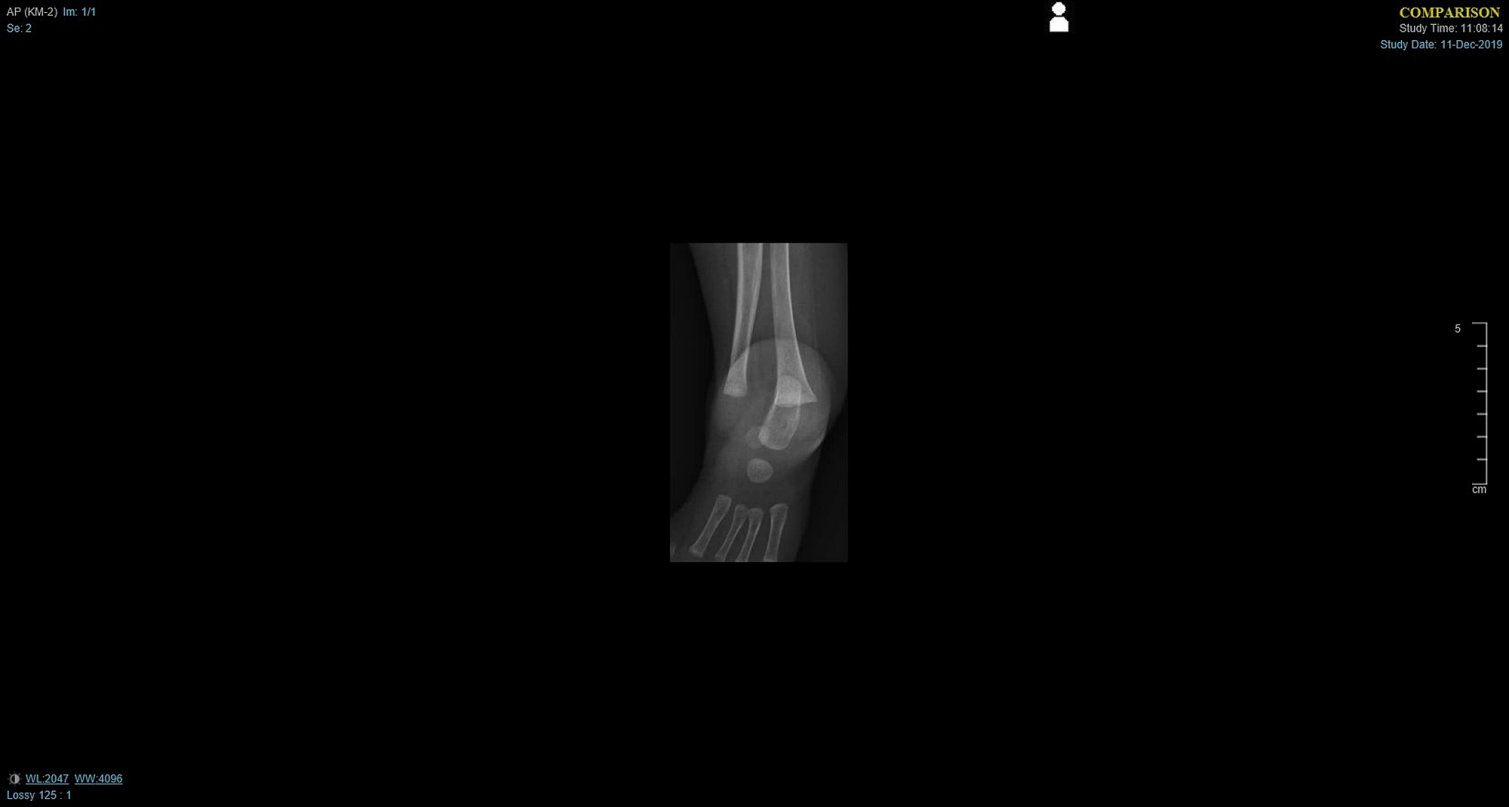
X-ray showing an underdeveloped left distal tibia and left distal fibula hypertrophy

- An X-ray showed an underdeveloped left distal tibia and left distal fibula hypertrophy (Figure 2).

- Doppler showed intact both left posterior tibial and dorsalis pedis arteries.

Surgical interventions

**Figure 3:**
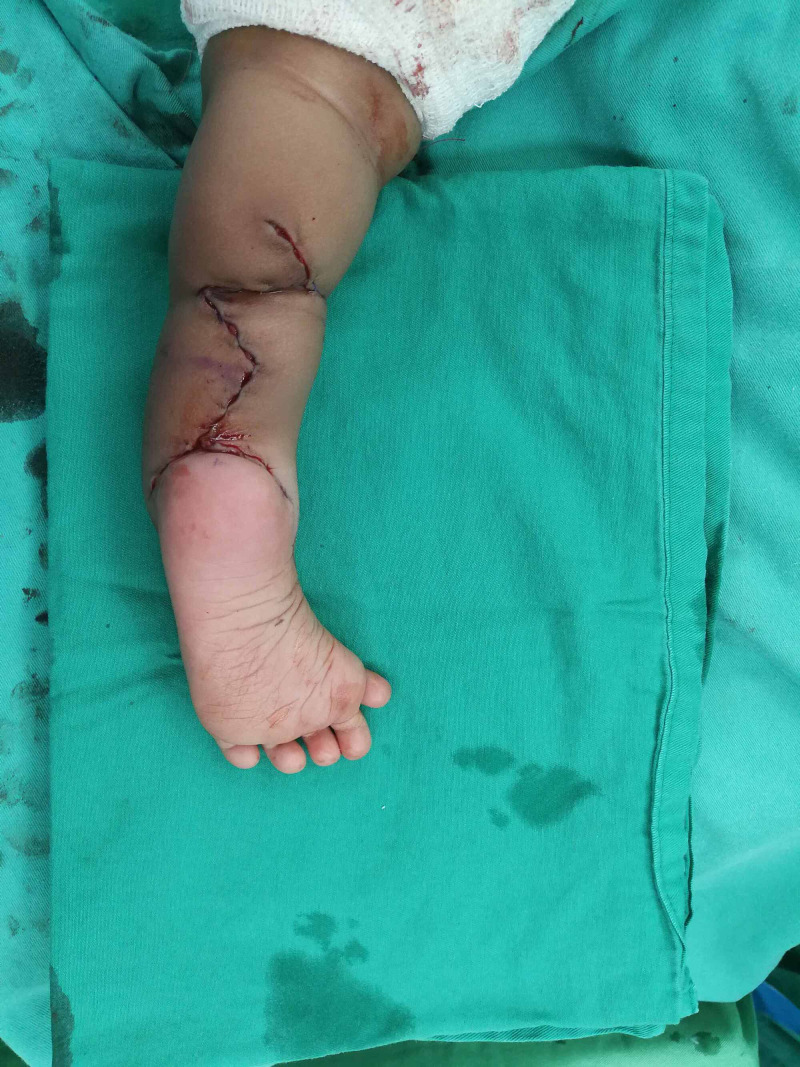
Left lower limb z-plasty reconstruction

Z-plasty was planned and performed to release the left tight ankle tendon aiming to increase the foot angle, as shown in Figure 3.

## Discussion

Embryonic mesodermal connective tissue gives origin to all connective tissues in the body, including cartilage and bone. Balanced endochondral and intramembranous ossification will result in average shape and growth of the long bones. Lower limb development involves complex and precise gene interactions that control bones' positional development [10]. The variety of congenital lower extremity shortening is wide-ranging and can include any leg bones and joints. In most cases, the definitive cause of long bone deficiency is unknown. However, some factors, such as muta­tions or teratogens/teratogenic drugs and early compromise of blood supply can cause impairment of mes­enchymal condensation and slow, or even abort the onset of chondrification process and cause leg length discrepancy associated with severe permanent morbidity related to abnormal weight-bearing and compro­mised ambulation. Tibial hemimelia or tibial deficiency is uncommon and markedly less frequent than the fibular variant [10, 11]. Its incidence is approximately about 1 per 1 million live births, with 0.8% occurring in monozygotic twins (about 1 case per 125 million) [4]. Usually, the clinical spectrum showed a pronounced family history of affected patients [9]. In literature, there are few recorded cases of tibial hemimelia in twins, specifically in identical or monozygotic twins. In 2003, Dayer and Kaelin published a case of tibial hemimelia in monozygotic twins, while in 2010, Leite et al. reported a case of tibial hemimelia in one of the identical twins [4]. Generally, there are no recorded cases, percentages, or data in the literature, about incidence in one of the dizygotic twins, with no family history of tibial hemimelia, which is against usual presentation.

Clinically, tibial hemimelia was classified into four types according to Jones classification [12], which was based on radiological criteria, but most recently, Paley published a new classification based on progressive deficiency patho-anatomy and as such serves to guide reconstructive options [2]. Tibial hemimelia may present as solitary disorder (unilateral or bilateral) or be a part of more complex malformation syndromes [7, 8]. Surgical treatment is varying according to the type and degree of deformity. Reconstructive interventions are still limited but significantly improved over the last decades. Other options include ankle arthrodesis, tendons lengthening or transpositions [13], and amputation, which is likely performed with a prosthetic foot, especially for the severely affected limb.

## Conclusions

Tibial hemimelia is a rare variety of congenital lower extremity shortening, and is very infrequently reported in the literature, especially tibial hemimelia in twins, with no reported cases in one of the dizygotic twins to normal parents without a family history of this deformity. In conclusion, some odds that may happen against usual presentation should be considered to elicit possible underlying causes and risk factors.
